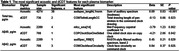# Exploring digital cognitive measures as non‐invasive biomarkers: Association with AD plasma biomarkers

**DOI:** 10.1002/alz.089047

**Published:** 2025-01-09

**Authors:** Huitong Ding, Cody Karjadi, Sherral A. Devine, Ashita S. Gurnani, Preeti Sunderaraman, Jesse Mez, Lindsay A. Farrer, Rhoda Au, Honghuang Lin

**Affiliations:** ^1^ Boston University Chobanian & Avedisian School of Medicine, Boston, MA USA; ^2^ Boston University Chronic Traumatic Encephalopathy Center, Boston University Chobanian & Avedisian School of Medicine, Boston, MA USA; ^3^ UMass Chan Medical School, Worcester, MA USA

## Abstract

**Background:**

Plasma amyloid and tau levels are known to be associated with the risk of Alzheimer’s disease (AD). In recent years, digital cognitive assessment has been increasingly recognized as a potential tool for long‐term cognitive monitoring. This study aims to explore the potential of digital cognitive metrics as efficient and low‐cost alternatives to plasma biomarkers.

**Method:**

This study included participants from the Framingham Heart Study who had either their spoken responses to neuropsychological examinations digitally recorded or had completed the digital Clock Test (dCDT), along with plasma biomarker measurements taken within five years. Simoa™ Tau 2.0 Kit and INNO‐BIA plasma Aβ were used for tau, and Aβ40 and Aβ42 measurement, respectively. The study analyzed digital cognitive metrics, comprising 65 acoustic features extracted via OpenSMILE software and 101 features derived from the dCDT. Linear regression models, adjusted for age and sex, were employed to examine the association between these digital cognitive metrics and plasma total tau, Aβ40, and Aβ42 levels.

**Result:**

This study included 4274 participants (mean age 57±14 years old, 53.7% women). Table 1 shows the top digital cognitive metrics associated with plasma biomarkers. Notably, plasma total tau was associated with 38 acoustic features. The most significant association was observed between audspec_lengthL1norm (sum of the auditory spectrum) and plasma tau (beta=‐0.09, SE = 0.03, P<0.001). Among the dCDT features, COMTotInkLengthCC, which represents the total drawing length of pen strokes in the command clock drawing, showed the most significant association with the plasma total tau level (beta=‐0.13, SE = 0.06, P=0.023).

**Conclusion:**

Our results demonstrate an association between acoustic and digital clock drawing with plasma total tau, which is consistent with previous research that finds that cognitive performance is linked to AD tau pathology. These results highlight the promise of these digital cognitive metrics as an easily scalable, low‐cost screening tool for those at high risk for AD. Future investigations to test the robustness of digital cognitive metrics as a surrogate screening measure will be critical in tackling AD pathology.